# Hepatic hydatid cyst – diagnose and treatment algorithm

**DOI:** 10.25122/jml-2018-0045

**Published:** 2018

**Authors:** Cristian Botezatu, Bogdan Mastalier, Traian Patrascu

**Affiliations:** 1. “Colentina” Clinical Hospital, General Surgery Clinic, Bucharest; 2. “Carol Davila” Medical University, Bucharest; 3. “Dr. I. Cantacuzino” Clinical Hospital, General Surgery Clinic, Bucharest

**Keywords:** liver hydatid cyst, surgical treatment, minimally invasive methods

## Abstract

**Introduction:** the hydatic disease, caused by the larvae of Echinococcus granulosus, is a serious disease, potentially lethal, which can be found anywhere in the world, but especially in endemic areas such as the Mediterranean Basin, Australia, New Zealand, North Africa, Eastern Europe, the Balkans, Middle East and South America. The hydatic cyst is mainly found in the liver (75% of the cases), being asymptomatic in most cases and discovered accidentally on a routine abdominal ultrasound or an ultrasound performed for diagnosing other pathologies. The hepatic hydatid cyst therapy is multimodal, including medical, surgical, and, lately, minimally invasive techniques.

**Materials and methods:** 88 patients were diagnosed with liver hydatid cyst at the General Surgery Clinic of the Colentina Hospital in Bucharest where they were admitted from January 2014 to July 2017. Data collection was realized by consulting the patients’ observation sheets, followed by organizing a database of clinical, paraclinical and treatment parameters. Age, gender, place of origin, year and duration of admission, symptoms and signs at admission, paraclinical serological tests relevant for liver function and E. granulosus infection, imaging investigations performed and their results, type of treatment received and post-treatment progress with the complications that occurred were taken in account.

**Results:** some of the results of the study showed some differences comparing to the data from specialty literature, the possible causes being the small number of patients, the paraclinical examinations that were not sufficiently detailed to allow the study of a phenomenon in all its complexity, the lack of information from the patients’ first presentation to a doctor or from their previous admissions.

**Conclusions:** patients with hepatic hydatid cyst form a heterogeneous group, semiology being poor and unspecific. Among the laboratory examinations, eosinophilia is a sign of concern but is present in less than half of the patients. Imaging findings are the basis for the diagnosis of hepatic hydatid cysts. Surgical treatment remains the “gold standard” in therapy, but minimally invasive methods with high applicability, less frequent complications and lower hospital requirements are starting to gain ground.

## Introduction

The hydatic disease is a severe, potentially lethal disease caused by Echinococcus granulosus larvae. The infection with E. granulosus should be seen as a challenge both from a medical and economic point of view [1].

In Romania, the incidence of this pathology is increasing, with 5-6 cases per 100.000 inhabitants each year [2].

**Figure 1:**
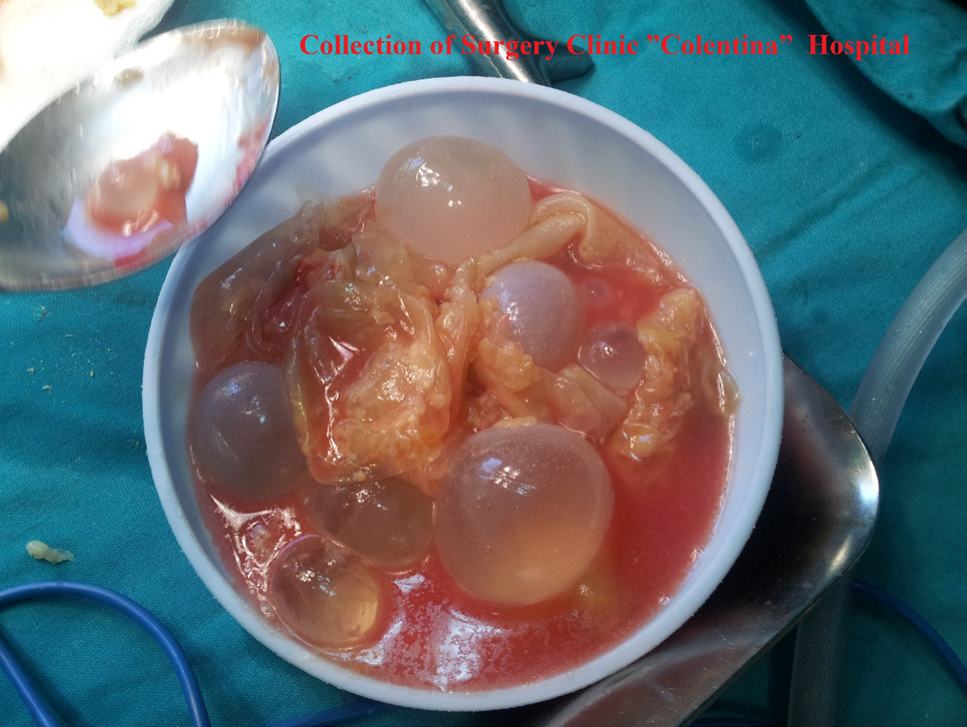
Hydatic cyst’s constitutive parts

**Figure 2:**
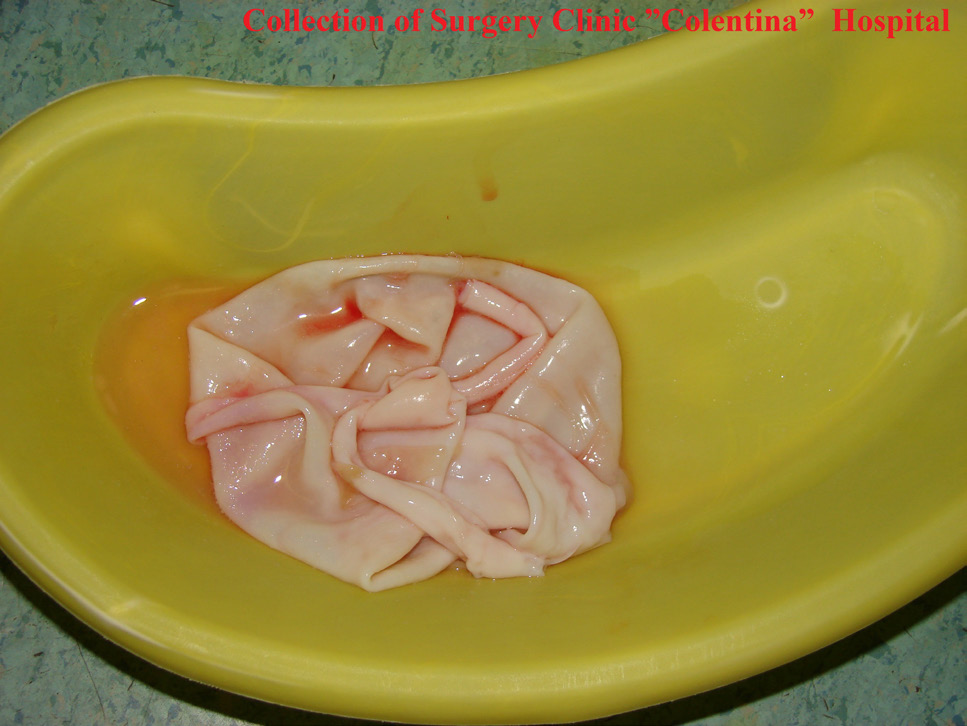
Proligere membrane

E. granulosus is a hermaphrodite flatworm with three stages of development. The structure of the cyst is usually made of three components: the pericyst, made of the host’s inflammatory tissue, the exocyst and the endocyst, where the scolecs and the proligere membrane are produced [3,4] (Figures 1, 2).

The hydatic cyst occurs by accidental infection of the human with the eggs of Echinococcus granulosus, followed by the development of the larvae, most commonly in the liver (50-70% of cases), and less commonly in the lungs, spleen, kidneys and brain [5-7].

**Figure 3:**
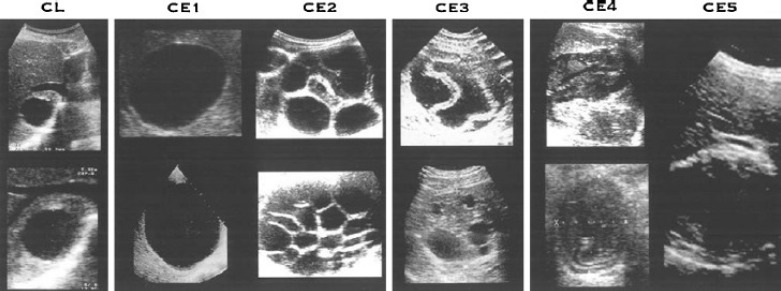
Brunetti E. Echinococcosis Hydatid Cyst 2015. Available from: http://emedicine.medscape.com/article/216432-overview.

At this time, the WHO-IWGE classification sets both the staging of hepatic hydatid cysts based on the ultrasound aspect, and the therapeutic attitude depending on this staging (Tables 1 and 2) (Figure 3) [8].

The therapeutic attitude towards hepatic hydatid disease includes the medical treatment, surgical treatment, endoscopic interventional treatment, as well as the subsequent minimally invasive methods.

Regarding the classical surgical treatment, mortality is around 0.9-3.6% and the recurrence rate is around 11.3% in the first 5 years [9].

The classical surgery procedures used for the treatment of the hepatic hydatid cyst are divided, according to their attitude towards the pericyst, into procedures that do not involve pericyst resection (cystectomy) and procedures involving pericyst resection (partial pericystectomy, pericystoresection, hepatectomy). They are associated with procedures that should treat the remaining cavity: external drainage with a drain tube, bipolar drainage of the cavity and the main bile duct, padding, omental plombage, drainage of the cavity by anastomosis with the stomach/jejunum, pericysto-biliar drainage. It should also be mentioned that hepatic transplantation might be a treatment option when at least 25-30% of the total hepatic parenchymal volume cannot be saved, or in the case of para- or post-hydatic hepatic cirrhosis [10]. The opening of the cystic cavity must be preceded by the inactivation of the parasite with a hypertonic saline solution, ethyl alcohol, hydrogen peroxide or Albendazole. It is also necessary to isolate the cyst from the rest of the peritoneal cavity, either by wrapping the adjacent areas with dressings soaked in anthelmintic substances or by applying adherent cones to the cyst using the icing technique or suction [11]. Resolving the remaining cavity is the primary challenge of the open surgical approach. In a study belonging to Mousavi et al., it is concluded that omental plumbing is superior to the drainage of the remaining cavity as it reduces the risk of seeding the peritoneum with germs [12].

**Table 1:** WHO-IWGE classification of the hydatid cyst

Stage	Echographic aspect according to WHO-IWGE Classification
CL	Anechogenic uniloculated cyst, with no echoes or internal sepsis
CE 1	Anechogenic cyst, with fine echoes inside, representing the hydatic sand - active cyst
CE 2	Cyst with multiple septums at the interior, giving it a multivesicular aspect or “honeycomb” aspect,with a uniloculated primary cyst - active cyst
CE 3	Uniloculated cyst with decolated proligere membrane (“waterlily sign”) (CE3a) or daughter vesicles associating hypo/hyperechogene images (CE3b) - cyst in transition phase
CE 4	Cyst with mixed content, hypo/hyperechogenic, without daughter vesicles - “wool clew” aspect-cyst in the degenerative phase
CE 5	Cyst with partial or totally calcified wall - inactive cyst

Laparoscopic interventions are primarily suited for cysts located superficially on the anterior surface of the liver without communicating with the biliary tree [11]. They can also be attempted in the case of multiple cysts (but fewer than three). The types of interventions that may be performed by laparoscopy are pericystotomy with cystectomy, partial or total pericystectomy, hepatic segmentectomy. It should be specified that laparoscopic liver resections are practiced with restrain, although mortality is around 1% [13]. During laparoscopic interventions, there is a higher risk of intraperitoneal hydatid fluid loss with the occurrence of secondary hydatidosis [9]. Haito et al. recommend that conservative operations should be performed laparoscopically, such as endocystectomy or total cystectomy, that allow the dissection at the level of the pericyst. He concludes that laparoscopic intervention is easier in small cysts (less than 6 cm) with superficial localization and in a more advanced stage of development [14]. The contraindications of laparoscopy are cyst rupture in the biliary tree, central cyst localization, cystic dimensions over 15 cm, thickened or calcified cystic walls [9].

**Table 2:** Therapy protocol for hydatid cyst

Stage	Size	First-option treatment	Alternative treatment
Refusal of intervention or contraindications for invasive treatment		ABZ (6 months)	
CE1, CE3a	Small	Only ABZ (6 months)	PAIR + ABZ (1 month)
	Medium	Surgical treatment + ABZ (1–6 months)	PAIR + ABZ (month)
	Large	Surgical treatment + ABZ (1–6 months)	MoCaT + ABZ (1 month)
CE2, CE3b	Small	Only ABZ (6 months)	MoCaT + ABZ (1 month)
	Medium	Surgical treatment + ABZ (1–6 months)	MoCaT + ABZ (1 month)
	Large	Surgical treatment + ABZ (1–6 months)	MoCaT + ABZ (1 month)
CE4, CE5	Any diameter	“Watch-and-Wait” attitude	“Watch-and-Wait” attitude
Complicated cysts, no matter what stage	Any diameter	Surgical treatment (+/- interventional endoscopy in case of rupture into the biliary tract)+ ABZ (6 months)	Surgical treatment in case of rupture; Percutaneous drainage in case of infection + ABZ (1 month)

The interventional endoscopy includes stenting on the main bile duct, Endoscopic Retrograd Cholangiopancreatography (ERCP), endoscopic sphincterotomy.

The minimally invasive techniques used in hepatic hydatid cyst treatment are PAIR, PAIRD, Modified Catheterisation Technique (MoCaT) or Percutaneous Evacuation (PEVAC). The PAIR technique (puncture, aspiration, injection of 95% ethanol solution or hypertonic saline solution, re-aspiration) is applicable to the hepatic hydatid cyst in stages CE1, CE2, CE3. The indications are: cyst with daughter vesicles +/-, detached proligere membrane, multiple cysts if accessible to puncture, superinfected cyst, patients refusing surgery, post-surgical relapse, patients with a surgical contraindication, patients not responding to drug therapy, pregnancy. Contraindications: non-cooperative patients, cysts that can not be punctured, inactive/calcified cysts, cysts that communicate with the biliary tree. The increased risk of secondary hydatidosis requires careful monitoring by postoperative serologic and imaging tests [15]. PAIRD (D=drainage) is a variant of PAIR that associates the insertion of an intracystic catheter at the end of the procedure. The cavity is irrigated with saline solution and drained for 24 hours. In case of cysts that communicate with the biliary tree, multivesicular cysts or cysts with content that cannot be suctioned, the PEVAC/MoCat (modified catheterization technique) can be used if the PAIR technique is not recommended. This technique involves inserting a 14F catheter into the cystic cavity, which will allow the evacuation of the solid content (daughter vesicles and endocyst) by successive injections and aspirations of an isotonic saline solution. The drainage tube is removed when the drainage is less than 10-15 ml/24 hours.

## Materials and Methods

Between January 2014 and June 2017, 88 patients diagnosed with hepatic hydatid cyst were admitted and treated at the General Surgery Clinic of the “Colentina” Hospital in Bucharest. The following parameters were taken into consideration: age, gender, place of origin, year and duration of admission, symptoms and signs at admission, serological and paraclinical investigations relevant to liver function and E. granulosus infection, the performed imaging investigations and their results, the received treatment and post-treatment evolution and complications.

Of the total number of patients enrolled in the study, 50 were female and 38 male. The age groups with the most representatives were 30-39 years and 40-49 years. The number of female patients was higher in the 30-39 and 40-49 age groups. Over half of the female patients were between 30 and 49 years of age. Male patients were distributed relatively evenly between 18 and 69 years of age.

More than half of the patients lived in rural areas. A higher incidence of hepatic hydatid cyst in rural patients may be explained by more frequent contact with herbivorous animals (sheep, goats, and others) that allow the E. granulosus life cycle to end with the perpetuation of the infection to dogs (the definitive host) and thus reach humans. However, the increased number of dogs in the urban environment (with or without an owner) makes the difference between the two environments not to exceed 6%.

The majority of the cases of hepatic hydatid cyst in this study were admitted in 2015, mentioning the fact that only the cases treated at the first visit were taken into account.

Regarding the symptomatology, the most commonly reported symptom was spontaneous or palpatory pain in the right hypochondrium. Other frequent manifestations were: asthenia and jaundice/slight jaundice.

Before surgery, liver function was investigated in all patients. Using the values of the two enzymes, the Ritis coefficient (AST/ALT, with normal values between 0.7 - 1.6) can be calculated.

Hepatic cytolysis syndrome, investigated through serum transaminases (AST and ALT), was most often identified. Excretory biliary syndrome or cholestatic syndrome was evaluated by measuring the serum alkaline phosphatase and serum bilirubin values. It was the least detected. Coagulation tests were used to investigate the hepatic insufficiency syndrome and were modified in 31 patients.

Over half of the patients presented inflammatory syndrome with plasma fibrinogen values above normal (200-400mg/dl).

Most of the patients were hospitalized, the symptoms investigated and treated at first in the Clinic of Parasitology. Having already had the results of previous paraclinical investigations, it was considered necessary to carry out immunological investigations in only 15% of patients. The serum IgG anti-Echinococcus granulosus antibody value was detected (it is considered positive when above 1,1 MU). Only one patient showed negative values in the immunological tests.

All patients received an abdominal ultrasound scan. Only 23.86% of patients required additional CT imaging. Many patients also required other imaging investigations, such as a chest X-ray, needed for the anesthesiologist’s evaluation. Also, in the imaging investigations we can include 3 cases of cholangiography (percutaneous transhepatic cholangiography in two cases and one case of cholangiography through a Kehr tube), and multiple cases of ERCP for diagnostic and/or therapeutic purposes, pre- or postoperative. Following the ultrasound examination of the entire group of patients, it was possible to collate a general distribution of cystic stages according to the WHO classification. More than half of the patients were treated for cysts in the CE3 stage. Nearly one-quarter of the study group presented cysts in the CE1 stage. Multiple liver cysts were met to the same extent as CE4 and CE2. The CE5 stage was the rarest.

A large percentage of CE1 and CE3 cysts with surgical treatment indication have been identified, although, at less than 5 cm diameter, the drug treatment as the only therapy is the primary intention. 10% were inactive cysts, but they needed to be treated, although the treatment recommendation in this case is a “watch-and-wait” approach. However, the patients included in the study group had characteristic symptoms (right upper quadrant pain on palpation and spontaneous fever and so forth) even under treatment with Albendazole.

In over half of the cases, the hydatid cyst was located at the level of the right lobe. Multiple cysts located in both hepatic lobes were met in 9% of cases.

Regarding the hepatic segments, the VII-th, the VIII-th and the VI-th were most often involved, followed by left segments III and IV, with 12% each. The most rarely involved was segment I. In most cases, two segments of the liver were involved.

For multiple cysts, the treatment was individualized (according to stage and size) for each cyst. Of the entire study group, 82% had cysts over 5 cm diameter, which excludes the possibility of single drug treatment (except for inactive and asymptomatic cysts). Only 18% of patients had cysts smaller than 5 cm, but in most of these cases, the invasive and minimally invasive treatment decision was justified by being in a CE2 or CE3 stage.

In the investigated group of patients, a majority of 89% presented uncomplicated cysts. In the other 11% of cases, the hepatic hydatid cysts were complicated by biliary fistula or superinfection. Five patients developed the fistula with the biliary tree as the only complication. There were 2 patients who, associated with the biliary tree communication, presented the following: angiocholitis, superinfection or both superinfection and pneumobilia.

Open surgical interventions prevailed (48 cases), followed closely by the minimally invasive ones (36 cases). There is no evidence of laparoscopic interventions. The most commonly used open-surgery procedure was cystectomy with partial pericystectomy (Lagrot operation), which was performed in 88% of patients. In 10% of patients, cystectomy was performed with the pericyst being left in place and the drainage of the remaining cavity. The healing of this cavity is dependent on the quality of drainage and the existence of biliary fistulae.

Of the minimally invasive treatment techniques, the PAIR technique and the modified version (MoCat or PEVAC) were used. MoCat type intervention was predominantly used, due to the fact that this technique extended its indications to cysts with biliary fistula and it allows the removal of solid hydatid material. In 3 cases, the completion of the MoCat procedure was impossible and one of the following three steps was taken: conversion to open surgery, timing of the final alcohol time due to a biliary fistula or simple inactivation with 30% NaCl.

In one patient with multiple cysts in the CE3 stage and biliary fistula, two minimally invasive interventions, PAIR and MoCat, were required.

## Results

Regarding complications, there were two complicated cases after the minimally invasive treatment and 12 after open surgery. Among the most common complications were prolonged biliary drainage from the remaining cyst cavity, due to the existence of a fistula between the cavity and the biliary tract, and infection. The difficulties that occurred during the procedure weren’t considered, which led to the impossibility of practicing minimally invasive treatment and forced the conversion to open surgery.

The larger number of patients with prolonged biliary drainage after open surgery can be explained by the fact that classical techniques mainly deal with complicated cysts with fistula, that would not allow minimally invasive treatment. Also, large incisions and the presence of drainage tubes over extended periods of time predispose to infection.

An average of the number of days of hospitalization based on the surgical treatment received was calculated. The cases treated with Albendazole as single therapy were not taken into consideration. An average longer hospitalization period was obtained after open surgery treatment (33.6 days). It is expected that minimally invasive interventions will require fewer days of hospitalization as they are less traumatic (incisions limited to the abdominal wall and the use of local anesthesia). A value of 11.76 days of hospitalization was obtained for minimally invasive techniques.

In this study group, the most common post-procedural complication was the prolonged biliary drainage, determined by the persistence of a residual cavity-bile tree communication. There were 3 cases of cholangitis (grade IIIa according to the Clavier-Dindo classification) that required antibiotic treatment and decompression of the biliary tract by ERCP with endoscopic sphincterotomy and, in one case, with the extraction of hydatid material. Also, post-surgical infection was encountered in 3 cases (grade II according to Clavier-Dindo classification). Infections are promoted by long immobilization in bed, the incision of the protective tegument and the presence of the drainage tubes over long periods of time. There have also been two cases of post-detubation anaphylactic shock (grade IVa according to the Clavier-Dindo classification) and a case of papillary stenosis (grade IIIa according to Clavier-Dindo classification).

Of the total number of patients, 71 showed favorable post-surgical development. Three of them were categorized as having a steady evolution because they only received medical treatment. In the category of slow favorable evolution, we included patients (8 cases) with a longer period of hospitalization (28-56 days), in which the closing of the remaining cavity was problematic due to the presence of biliary fistula and prolonged biliary drainage. This category also involved the case of a patient in which the MoCAT procedure could not be performed in a single operating sequence and it was necessary to complete it later, after the biliary drainage stopped. There were 6 cases of unfavorable evolution for which additional intervention was performed by ERCP with sphincterotomy with or without extraction of hydatid material or antibiotic treatment (grade IIIa according to Clavier-Dindo classification).

One of the patients that were treated using minimally invasive techniques (PAIR or MoCat) needed two interventions of this kind for two CE3 cysts located in the left lobe. In three patients, surgery was not performed because the anesthetist’s evaluation contraindicated it or because of the patient’s refusal, so single therapy with Albendazole was continued.

Following the above, we can state that the study group presented heterogeneity in terms of age and gender. Women were more numerous than men (50 vs. 38), and nearly half of the patients were in the 30-49 age group.

From the point of view of the symptomatology, the majority were those who did not show clinical manifestations. Paraclinically, 39% of patients experienced eosinophilia, a much more specific change for parasitic infection than hepatic syndromes or inflammatory syndrome that were previously considered. The imaging investigation represented by an abdominal ultrasound was the one that ultimately linked the serological modifications and symptoms to the diagnosis of hepatic hydatid cyst. Also, according to the imaging, it was possible to choose the optimal therapy (depending on the stage and the cysts’ dimensions).

The treatment options were mostly surgical (classic and minimally invasive) in accordance with the stage (very common CE3 and CE1) and the size (mostly over 5 cm) of the cyst. Only in 10% of the cases the interventions were performed for inactive cysts (CE4 and CE5), in which the “watch-and-wait” attitude can be adopted.

If the patient’s condition did not allow general anesthesia, surgery was postponed or replaced with drug therapy.

The post-treatment evolution was favorable in 81% of the cases, a fact that is entirely expected when considering a benign condition with multiple therapeutic options and an ongoing development.

Comparing the results obtained with those in literature, both similarities and consistent differences were found. If women were more numerous than men in the study group, the general data denies the existence of a significant difference in the incidence in the two sexes. Also, the age most commonly diagnosed with hepatic hydatid cyst is 45-64 years, but in this case, the age group with the most patients was 30-49 years. From the paraclinical point of view, there are differences in the frequency with which eosinophilia is encountered (25% in the literature versus 39% in the present group) and the type of hepatic syndrome more common (cholestasis syndrome in the literature versus the cytolysis syndrome). The most frequent location was in the right hepatic lobe, same as in specialist literature. However, the frequency of complicated cysts was much lower in the study group (up to 50% in other studies vs. 11%). In terms of treatment, it is advisable to choose between medication, minimally invasive techniques and open surgery, all used depending on the stage and size of the cyst. Among the most commonly used open surgery procedures is the Lagrot cystectomy with partial pericystectomy, both in specialty literature and in our study group (88% of open surgery interventions).

## Conclusions

The lot was characterized by heterogeneity regarding the gender, age and environment of origin. There was a slight female predominance and an increased share of the 30-49 age group. The age range was between 18 and 84 years old, mentioning the fact that the Surgical Clinic is one for adults.

From the clinical examination, we highlight the high percentage of asymptomatic patients, which confirms once again the high frequency of accidental diagnosis in hepatic hydatid cyst. The most frequent symptom at patients’ admission is represented by pain located at the level of the right hypocondrium, a nonspecific symptom. The fact that the results obtained are for cases referred for surgical treatment and may differ from those obtained at the first diagnosis (frequently in another clinic) and prior to treatment with Albendazole must not be forgotten.

Laboratory examinations revealed hepatic cytolysis syndrome most frequently with elevated ALT values (and in cases where additional hepato-biliary pathologies were not associated), inflammatory syndrome evaluated by a single parameter (fibrinogen) and eosinophilia (39% of cases).

As previously shown, there are several factors that can justify the clinical and paraclinical changes noticed besides the hydatid cyst. Among these, we can list associated comorbidities and the administered medication, diagnosis and treatment in other medical units, and prior treatment with Albendazole.

Imaging, especially the ultrasound examination, was the basis for pre-surgical characterization of cysts. Most of the cysts were CE3 and CE1 stages and, in terms of size, between 5 and 10 cm diameter. The right lobe has been affected in more than half of the cases.

Regarding the minimum invasive treatment, the MoCAT and PAIR techniques were used. Among the open surgical procedures, in 88% of the cases, the Lagrot technique was used. The comparison between classic and minimally invasive surgical treatment revealed fewer complications and fewer necessary days of hospitalization in favor of the latter.

Summarizing, the following can be said: — patients with hepatic hydatid cyst form a heterogeneous group (taking into account gender, age, place of origin); — semiology is poor and unspecific; — among laboratory examinations, eosinophilia is a sign of concern that should place the hydatid liver cyst on the differential diagnosis list; — imaging, most commonly in the form of ultrasound examination, the easy, cheap and non-irradiation method is the basis of the diagnosis of hepatic hydatid cyst; — minimally invasive methods have high applicability, less frequent complications and shorter hospitalization; — the therapeutic solution for the hepatic hydatid cyst remains the attribute of general surgery, both by the still important role of classical and laparoscopic surgical techniques and by the ability of surgery to provide therapeutic assistance to cases treated through minimally invasive techniques.

## Conflict of Interest

The authors confirm that there are no conflicts of interest.
