# Correction to: Hepatic hydatid cyst – diagnose and treatment algorithm

**Published:** 2018

**Authors:** Cristian Botezatu, Bogdan Mastalier, Traian Patrascu

**Affiliations:** 1. “Colentina” Clinical Hospital, General Surgery Clinic, Bucharest; 2. “Carol Davila” Medical University, Bucharest; 3. “Dr. I. Cantacuzino” Clinical Hospital, General Surgery Clinic, Bucharest

## Abstract

In the original publication, the corresponding author field has been omitted. The correct corresponding author name is: Bogdan Mastalier. The original article is now corrected.

In the original publication J Med Life 2018; 11:203-9[1], the corresponding author field has been omitted. The correct corresponding author name is: Bogdan Mastalier. The original article is now corrected. We wish to apologise for any misunderstanding or inconvenience caused.
